# Estimation of HbA1c Levels in Transfusion-Dependent Thalassemia Patients in Comparison With Normal Healthy Individuals

**DOI:** 10.7759/cureus.73236

**Published:** 2024-11-07

**Authors:** Parmanreet Kaur Bhatti, Richa Ghay, Gursharan Singh Narang, Varidhi Thaman, Suneet Narang

**Affiliations:** 1 Internal Medicine, Sri Guru Ram Das Institute of Medical Sciences & Research, Amritsar, IND; 2 Physiology and Medical Education, Sri Guru Ram Das Institute of Medical Sciences & Research, Amritsar, IND; 3 Paediatrics, Sri Guru Ram Das Institute of Medical Sciences & Research, Amritsar, IND; 4 Internal Medicine, Maharishi Markandeshwar Institute of Medical Sciences, Mullana, IND

**Keywords:** diabetes mellitus, fasting blood sugar (fbs), hba1c, transfusion dependent thalassemic (tdt) patients, β-thalassemia

## Abstract

Introduction

HbA1c values used for diagnosing and treating diabetes can be affected by factors such as red blood cell lifespan, hemolysis, red cell transfusion, and the presence of minor Hb species like HbA2 and HBF in hemoglobinopathies like sickle cell disease, homozygous HbC disease, HbSC disease, and β-thalassemia. This study aims to compare HbA1c levels in transfusion-dependent thalassemia (TDT) patients and healthy individuals.

Materials and methods

This is a cross-sectional comparative study. This study comprises two population groups. The first group includes 35 TDT patients and the second group consists of 35 non-thalassemic individuals who were matched for age (±1 year), gender, and BMI (±1 kg/m^2^). The patients were selected from the pediatric outpatient department (OPD), thalassemia ward in the pediatric department, and medicine OPD. Written informed consent/assent was obtained from the participants. A 3 ml fasting venous blood sample for fasting blood sugar (FBS), HbA1c, and complete blood count (CBC) values was obtained in ethylenediaminetetraacetic acid (EDTA) vials on the scheduled blood transfusion day (pretransfusion samples). Samples were then sent to an in-house accredited lab for testing and analysis. HbA1c was performed using the high-performance liquid chromatography (HPLC) technique. Data was compared using a t-test. Qualitative parameters were compared between groups by X^2 ^square analyses. A multivariate linear regression model was used to explore the independent contribution of an individual predictor to HbA1c variability.

Results

In the study, 85.7% of patients with TDT had HbA1c levels in the diabetic range (>6.4%). In comparison, none of the control group patients had HbA1c values in the diabetic range. The mean HbA1c level was 6.94% in TDT cases and 5.3% in the control group, which was statistically significant (p < 0.001). Elevated FBS levels in the prediabetic range (>100 mg/dl, <126mg/dl) were observed in 25.7% TDT cases. All 35 controls had normal FBS levels (<100 mg/dl). No significant difference was found in FBS levels between cases (92.97 (±9.141) mg/dL) and controls (89.20 (±7.584) mg/dL) (p = 0.065). However moderately positive correlation exists between FBS and HbA1C (r =.470, p = 0.004) and between age and HbA1C (r = 0.335, p = 0.049).

Conclusions

The use of HbA1c as a screening tool for diabetes mellitus (DM) or assessment of glycemic control is inappropriate in TDT patients. The levels could be falsely elevated, as we found out in our study. In conditions where there is a mismatch between HbA1c and FBS levels, as seen in TDT patients, plasma glucose criteria should be used to diagnose diabetes. It is advised to use alternative indices such as fructosamine levels, glycated albumin, and continuous glucose monitoring.

## Introduction

HbA1c also known as glycated hemoglobin (Hb) is considered to be the biochemical indicator to track the glycemic control and risk of developing complications in patients with diabetes mellitus (DM) [1]. HbA1c estimation may be impeded by conditions that alter red cell half-life, e.g., hemolysis, hemorrhage, red cell transfusion, and the presence of minor Hb species like HbA2 and HbF. These Hb species may be increased in many hemoglobinopathies like sickle cell disease, homozygous HbC disease, HbSC disease, and β-thalassemia [2].

To obtain accurate HbA1c results, it's important to identify Hb variants, as they can affect outcomes. Laboratories should be cautious when reporting results if a variant is suspected. Manufacturers may need to update guidelines and testing methods to reduce errors. Any HbA1c result that contradicts clinical observations should be investigated further [3].

Thalassemia is an inherited blood disorder that involves inadequate production or absence of synthesis of one or more globin chains of Hb. The disorder leads to the destruction of many red blood cells, leading to brief time exposure to glucose [4]. The estimation of HbA1c levels is based on an RBC lifespan of 120 days, and diminished RBC survival tends to lower its levels [5]. In transfusion-dependent thalassemia (TDT) patients, the circulating Hb is that of donors. Moreover, the lifespan of RBCs is also reduced; therefore, caution should be taken to carefully assess and interpret HbA1c values in these patients [6].

A multicenter study across six Indian states, Maharashtra, Gujarat, West Bengal, Assam, Karnataka, and Punjab, assessed the prevalence of hemoglobinopathies among different caste and ethnic groups. The study found that the overall prevalence of β-thalassemia in India is between 3% and 4%, with state-specific rates ranging from 1.48% to 3.64% [7].

Beta-thalassemia is the most prevalent monogenic disorder in India, particularly in the Punjab region. In Punjab, the prevalence of heterozygous β-thalassemia is 3.96%, and β-thalassemia major requiring blood transfusion is 0.26%. The incidence of β-thalassemia major is 700-800 births per year [8].

There are few studies available on the erroneous nature of HbA1c measurement in patients with thalassemia. A multicountry survey including 14 centers involved in the International Network on Endocrine Complications in Thalassemia (ICET-A) network reports the prevalence of DM in β-thalassemia to vary from 9.7% to 29% and the overall prevalence of impaired fasting glucose (IFG) as 17.2% [9]. Another relevant study, a meta-analysis including 44 papers from PubMed, ScienceDirect, SpringerLink, Ovid, Web of Science, MEDLINE, and other databases estimates a very high prevalence of abnormal glucose metabolism in β-thalassemia major [10]. Our null hypothesis is that there is no significant difference in HbA1c levels between individuals with thalassemia and those without thalassemia.

## Materials and methods

This is a cross-sectional comparative study. This study comprises two population groups. The first group includes the TDT patients and the second group consists of non-thalassemic individuals who were matched for age (±1 year), gender, and BMI (±1 kg/ m2). The patients were selected from the pediatric outpatient department (OPD), thalassemia ward in the pediatric department, and medicine OPD. 

All TDT patients (TDT, thalassemia major) who willingly gave consent were recruited in this study. Certain patients such as those diagnosed with other hemoglobinopathies such as HbS and HbC disease, chronic kidney disease stage III (eGFR < 60 ml/min/1.73 m2), or any other major comorbidities (i.e., malignancies, heart failure of clinical stage NYHA ≥II, chronic hypoxemic states, chronic liver disease, chronic inflammatory states) were excluded from the study. Patients with any other clinical condition apart from thalassemia that could potentially affect hematopoiesis and/or erythrocyte turnover were also excluded.

Patients who visited the OPD for a general health checkup and consented to the study were selected as the second group, consisting of non-thalassemic individuals. They were asked to return on a fasting basis for a fasting blood sugar (FBS) test in the next few days. Those who returned and consented to the test were included in the study. However, those diagnosed with type 1 or type 2 DM were excluded from the study.

For this study, we enrolled 35 TDT patients coming to our hospital for bimonthly blood transfusions and 35 non-thalassemic individuals. A total of 35 TDT patients are enrolled for blood transfusion in the thalassemia ward in our institute. 

Patient recruitment

A written informed consent was obtained from all participants in their vernacular language (by both parent and minor). The study was carried out in accordance with the principles of the Declaration of Helsinki [11], and approval from Institute Ethics Committee was sought before enrolling the first participant. After enrolling the participants, a proforma with patient details including age (years), gender, height (m), and weight (kg) for calculating BMI (kg/m2), h/o smoking, and family history of type 2 DM was filled for all. Blood samples for Hb, HbA1C, and complete blood count (CBC) were collected. A 3 ml venous sample after overnight fasting for FBS, HbA1c, and CBC values was collected. Samples were sent to the institute NABL accredited lab for testing and analysis. HbA1c is performed in the lab by high-performance liquid chromatography (HPLC) technique.

A list of the patients was obtained from the thalassemia ward in the department of pediatrics. The patients were approached a day in advance and requested to come fasting for the test if possible. The patients arrived at the hospital around 8:30 am to 9 am. The study was explained in detail to the patients. A written informed consent/assent was obtained from the patients and their parents as appropriate. For both cases and controls between eight and 18 years, assent from parents was also obtained along with consent from participants. For participants above 18 years, only informed consent from them was obtained. Data collection forms (DCFs) were filled out for all participants. The DCF was given a unique patient identification number to maintain the confidentiality of records. Fasting venous blood samples were obtained in ethylenediaminetetraacetic acid (EDTA) vials on the day of the scheduled blood transfusion (pretransfusion samples). If the fasting condition was not satisfied at the time of enrollment, the sample was collected on the subsequent visit of the patient. Similarly, fasting blood samples were obtained from the control group recruited from pediatric and medicine OPD in the hospital. FBS values were classified according to the American Diabetes Association (ADA) criteria [12].

Data analysis

All statistical analyses were conducted using Epi Info 7 (Centers for Disease Control and Prevention (CDC), Atlanta, Georgia, USA). The Kolmogorov-Smirnov and Shapiro-Wilk tests were used to assess the normality of parameters among the participants. Continuous variables are expressed as mean ± standard deviation, while categorical variables are presented as percentages. To identify statistical differences between the two groups, a Student's t-test was applied for continuous variables and a chi-square test for categorical variables, with statistical significance set at a two-tailed p-value of <0.05. Pearson’s correlation was used to assess the relationship between blood parameters, HbA1c, and FBS levels.

## Results

Our study included 35 TDT patients and 35 age- and gender-matched healthy individuals. Both groups comprised 18 females (51.4%) and 17 males (48.6%). Table 1 illustrates that the age distribution is similar between cases and controls, with the majority of participants aged 11-20 years (60.00% of cases and 62.86% of controls). In the TDT patient group, 22.86% are 10 years or younger, and 17.14% are aged 21-30 years.

**Table 1:** Distribution of age and gender among TDT patients and healthy controls TDT: Transfusion-dependent thalassemia The data has been represented as N (count) and %. Chi-square test was applied to calculate the p-value

		Cases		Controls		χ^2^ value	p-value
		Number	Percent	Number	Percent		
Age group (years)	≤10	8	22.86%	7	20.00%	0.089	0.903
	11-20	21	60.00%	22	62.86%		
	21-30	6	17.14%	6	17.14%		
Gender	Female	18	51.43%	18	51.43%	NA	NA
	Male	17	48.57%	17	48.57%		

The gender distribution is balanced in both groups. The average age is 15.54 ± 4.90 years for the patients and 15.69 ± 4.8 years for the healthy individuals. A Chi-square test revealed no significant difference in average age between the thalassemia patients and healthy controls (p = 0.903), confirming a similar age distribution across the groups (Table 1).

Out of the 35 TDT patients, five exhibited HbA1c levels in the prediabetic range (5.7%-6.4%), while the remaining 30 had HbA1c levels in the diabetic range (>6.4%). Notably, none of the TDT patients had HbA1c levels classified as normal (<5.7%).

This study highlights that none of the TDT patients fall within the "normal" glycemic category, in stark contrast to 88.57% of healthy controls, who are classified as normal. Furthermore, a significant majority of thalassemia patients (85.70%) are categorized as diabetic, whereas no healthy controls fall into this category.

The mean HbA1c level in thalassemia patients is significantly higher at 6.93 ± 0.49 compared to the control group, which had a mean of 5.30 ± 0.35. The p-value of <0.001 indicates a statistically significant difference in glycemic control between the two groups (Table 2).

**Table 2:** Comparison of glycemic status between TDT patients and healthy controls TDT: Transfusion-dependent thalassemia; HbA1c: glycosylated hemoglobin The data has been represented as N (count)and %. Independent student t test applied

	Total	Cases		Controls	
		Number	Percent	Number	Percent
Normal	31	0	0.00%	31	88.57%
Prediabetics	9	5	14.30%	4	11.43%
Diabetics	30	30	85.70%	0	0.00%
Mean HbA1c(%)		6.93 ± 0.49		5.30 ± 0.35	
t-value, p-value		15.896, <0.001			

Among thalassemia patients, nine (25.70%) are classified as prediabetic, based on elevated FBS levels (>100 mg/dL and <126 mg/dL). In contrast, none of the healthy controls fall into this category. The remaining 26 TDT patients had normal FBS levels (<100 mg/dL), and none exhibited FBS levels in the diabetic range (>126 mg/dL). All 35 control subjects maintained normal FBS levels (<100 mg/dL).

The mean FBS level for thalassemia patients is slightly higher at 92.97 ± 9.14 mg/dL compared to the control group, which has a mean of 89.20 ± 7.58 mg/dL. However, the p-value of 0.065 indicates that this difference is not statistically significant. Therefore, there is no significant difference in FBS values between thalassemia patients and healthy controls, as the p-value exceeds the 0.05 threshold (Table 3).

**Table 3:** Distribution of Fasting Blood Sugar (FBS) Levels in TDT Patients and Healthy Controls TDT: Transfusion-dependent thalassemia; FBS: fasting blood sugar The data has been represented as N (count) and %. Independent student t test applied

FBS		Total	Cases		Controls	
			Number	Percent	Number	Percent
Normal	(<100mg/dl)	61	26	74.30%	35	100%
Prediabetics	(>100 mg/dl, <126mg/dl )	9	9	25.70%	0	0%
Mean FBS (mg/dL)			92.97 ± 9.14		89.20 ± 7.58	
t-value, p-value			1.879, 0.065			

**Figure 1:**
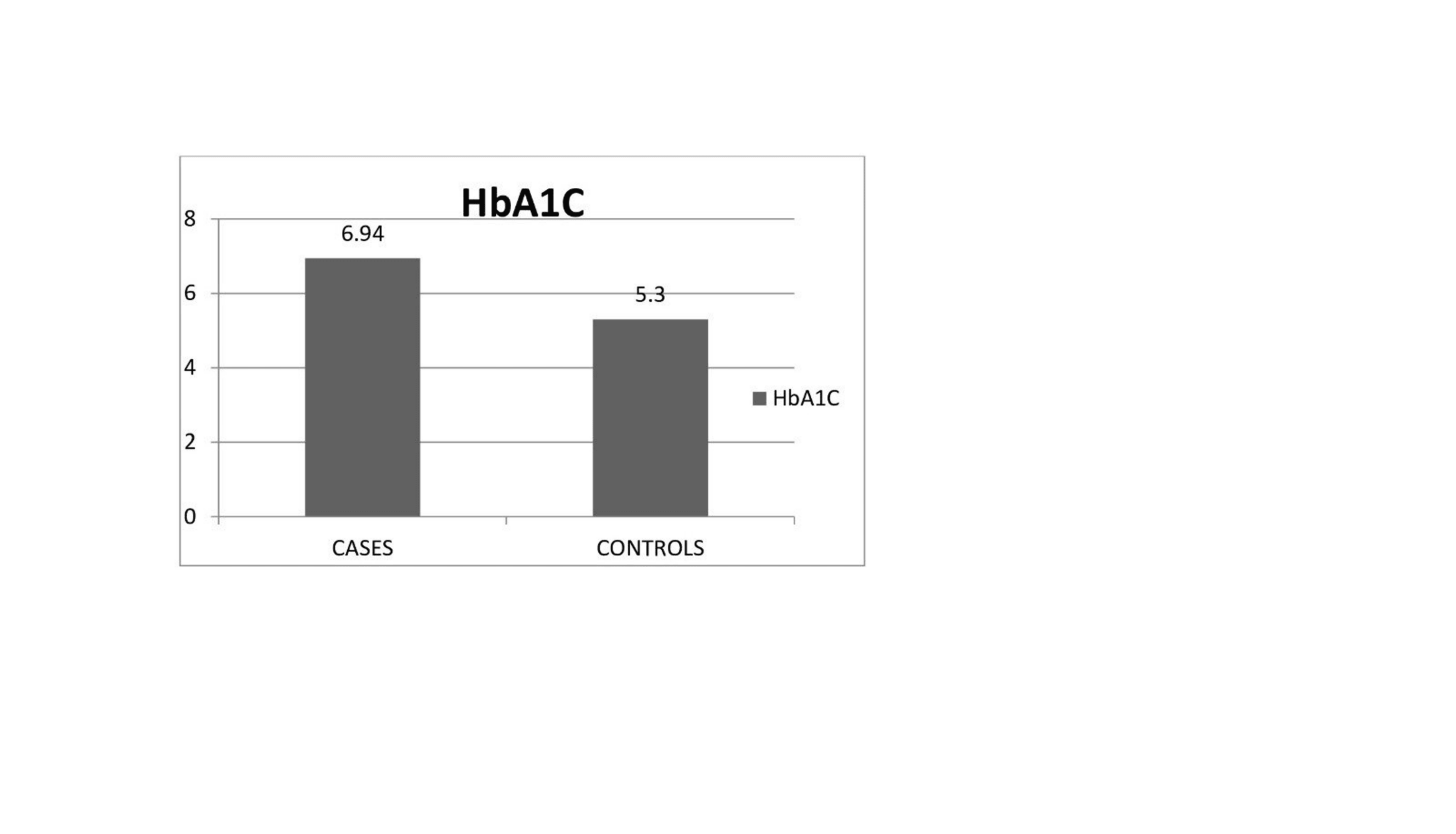
Mean values of HbA1c for cases and controls The data has been represented as mean ± SD value, 6.93 ± 0.495 for cases and 30 ± 0.35 for controls

The mean value of HbA1c in cases is 6.94%, while it is 5.3% in controls, as shown in Figure 1.

**Figure 2:**
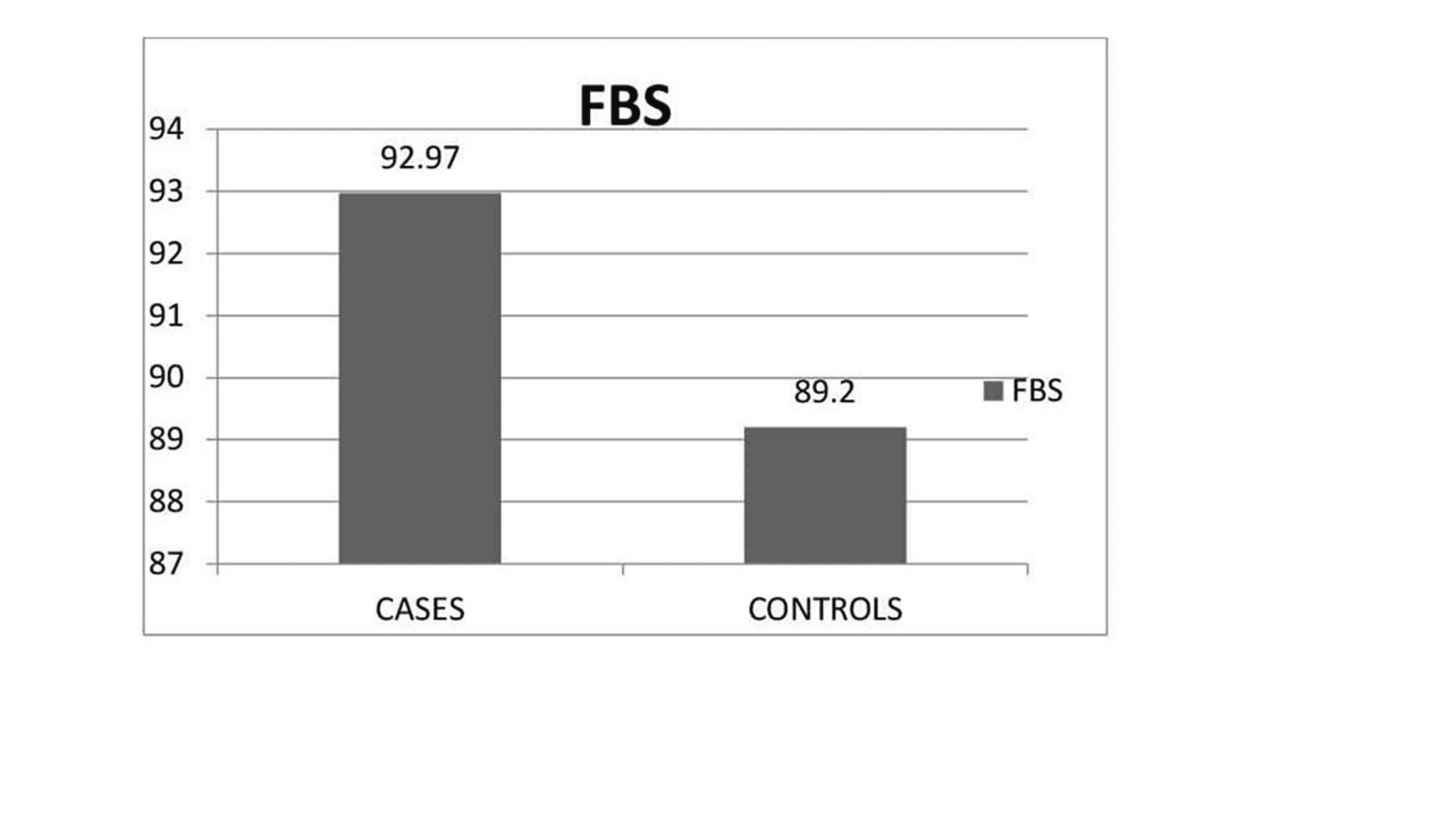
Mean values of FBS for cases and controls The data has been represented as mean ± SD values, 92.97 ± 9.14 for cases and 89.20 ± 7.58 for controls

The mean value of FBS in cases is 92.97 mg% and 89.2% in controls as shown in Figure 2.

Significant differences are observed in red blood cell (RBC) count, Hb, packed cell volume (PCV), and mean corpuscular volume (MCV) between thalassemia patients and controls (p < 0.001). All these parameters are lower in TDT patients compared to the control group. Specifically, the p-values for RBC count, PCV, Hb, and MCV are all below 0.001, indicating strong statistical significance.

In contrast, the mean corpuscular hemoglobin (MCH) does not show a statistically significant difference between the two groups, with a p-value of 0.118. However, mean corpuscular hemoglobin concentration (MCHC) is significantly higher in thalassemia patients compared to controls (p < 0.001). These findings may be attributed to factors such as chronic anemia, iron overload resulting from frequent blood transfusions, and endocrine complications commonly seen in TDT patients.

Refer Table 4.

**Table 4:** Comparison of hematological parameters between TDT patients and healthy controls RBC: Red blood cell; PCV: packed cell volume; MCV: mean corpuscular volume; MCH: mean corpuscular hemoglobin; MCHC: mean corpuscular hemoglobin concentration; Hb: hemoglobin p < 0.05 is statistically significant, p < 0.001 is highly significant. Independent student t test applied

Parameter	Cases	Controls	t Value	p-value
Hb (g/dL)	8.89 ± 1.12	13.07 ± 1.47	-13.374	<0.001
RBC count (×10^12^/L)	3.23 ± 0.4	4.61 ± 0.5	-12.773	<0.001
PCV (%)	26.44 ± 3.53	43.31 ± 5.24	-15.791	<0.001
MCV (fL)	82.97 ± 3.69	93.96 ± 10.87	-5.666	<0.001
MCH (pg)	27.1 ± 3.77	28.41 ± 3.15	-1.583	0.118
MCHC (g/dL)	33.32 ± 1.62	30.5 ± 2.48	5.624	<0.001

HbA1c levels are statistically significantly higher in thalassemia patients (6.94% ± 0.50%) compared to controls (5.30% ± 0.35%), with a p-value of <0.05. This finding contradicts our initial hypothesis that there would be no significant difference in HbA1c levels between individuals with thalassemia and those without.

To explore the factors contributing to the differences in HbA1c levels between the two groups, a Pearson correlation analysis was conducted. The correlation coefficient "r" was used to assess the strength of the association, where values close to one indicate a strong correlation, either positive or negative. The p-value was utilized to determine the significance of the results, with values of ≤0.05 considered statistically significant (Table 5).

**Table 5:** Correlation analysis of hematological parameters with fasting blood sugar (FBS) and HbA1c levels in TDT patients and healthy controls RBC: Red blood cell; FBS: fasting blood sugar; HbA1c: glycosylated hemoglobin; Hb: hemoglobin The Pearson correlation was applied

Group			Hb	RBC count	PCV	MCV	MCH	MCHC
Cases	FBS	r value	0.013	0.069	0.01	-0.200	0.005	0.061
		p-value	0.94	0.692	0.954	0.250	0.978	0.726
	HbA1c	r value	-0.106	-0.015	-0.052	-0.129	-0.156	-0.105
		p-value	0.544	0.931	0.768	0.461	0.371	0.547
Controls	FBS	r value	.352^*^	0.267	0.28	0.11	0.213	0.195
		p-value	0.038	0.12	0.104	0.528	0.220	0.261
	HbA1c	r value	0.143	.337^*^	0.274	0.032	-0.052	-0.120
		p-value	0.413	0.048	0.111	0.855	0.766	0.494

As shown in Table 5, within the control group, fFBS exhibits a significant positive correlation with Hb (r = 0.352, p = 0.038). Additionally, HbA1c levels in the control group show a significant positive correlation with RBC count (r = 0.337, p = 0.048). In contrast, no significant correlations were found between FBS or HbA1c and any hematological parameters in the TDT group.

## Discussion

HbA1c is the standard tool for monitoring glycemic control in patients with diabetes, at the same time we need to know the clinical scenarios that interfere by giving false results. DM is a common complication in people with TDT. 

Our study shows mean HbA1c values to be 6.94% in cases and 5.3% in controls. With these values, 85.7% of the cases were diabetic. The mean FBS value was 92.97 mg% in cases and 89.2 mg% in controls. According to FBS values, none of the TDT participants were diabetic, and only 25.7% had prediabetic values. A highly significant difference was seen in values of HbA1c in cases and controls. In comparison, no significant association was seen in FBS values in the two groups.

In our study, no TDT patient was found to be diabetic by FBS values and only 25.7% were found to be prediabetic. Our study shows very high diabetic prevalence with HbA1c values (85.7%), but none of the participants was diabetic by using FBS values. Only 25.7% of patients had their FBS values in the prediabetic range and 0% were diabetic.

The most significant finding of our study is the elevated HbA1C levels in TDT. The utility of the use of HbA1c in TDT has its limitations as thalassemia, blood transfusions, and shortened RBC lifespan interfere with its values and interpretation [13]. A similar increase in the HbA1C levels was also demonstrated by a study based in Delhi, India, by Gomber according to which HbA1c was significantly higher in cases (7.10% (±0.47%)) than in controls (5.15% (±0.19%)) (p < 0.001). This study also found higher HbA1c levels in those with abnormal glucose tolerance (oral glucose tolerance test) (7.34% (±0.57%)) as compared to those with normal glucose tolerance (7.05% (±0.43%)) (p = 0.05) [14]. These findings strongly correlate with our study.

A study by Choudhary et al. documented elevated HbA1c results using HPLC in thalassemia major regardless of glycemia. In this study, all 12 subjects had elevated HbA1c. HbA1c was 7.4% in three patients with normal glucose tolerance, 7.33% in three patients with prediabetes, and 8.16% in six patients with DM [15]. Our study also uses the HPLC method for the measurement of HbA1c levels and shows similar levels of HbA1c in TDT patients. All TDT participants in our study had elevated HbA1c levels, 14.3% in prediabetic and 85.7% in diabetic range values according to the ADA. Therefore, the use of HbA1c as a screening tool for DM in this population is inappropriate.

There are contrary reports regarding the effect of recent blood transfusion on HbA1C levels. Some recent studies suggest that dilutional effects because of the volume of transfused RBCs in a patient without diabetes can result in falsely decreased HbA1c [16]. Previous studies have shown that exposure of RBCs to a high glucose concentration of storage medium results in falsely elevated HbA1C levels. The results of a study at the University of Vienna suggest a minor increase in HbA1c concentration when blood was stored for 42 days [17]. These conflicting reports make it difficult to interpret the HbA1c results in TDT patients.

The prevalence of impaired glucose tolerance (IGT) and insulin-dependent DM (IDDM) in adolescents and young adults with TDT, mainly treated with deferoxamine (desferrioxamine), varies in different series from 0% to 17% [18]. A study conducted in Chhattisgarh, India, based on the glycemic status in children with β-thalassemia major states the mean value of HbA1C in normal children to be 4.79%, whereas in prediabetic children, the mean value of HbA1C was 6.13%. A total of 11.18% children in this study were prediabetic [19].

All of the TDT participants were being given chelation therapy because transfusion-related iron overload is a critical complication in TDT patients. Though chelation is now being widely used, patients still carry extrahepatic iron burdens. Cumulative iron overload and natural aging, combined with insulin resistance and beta cell destruction, together contribute to the development of DM in these patients. A study by Merkel et al. aimed at determining whether iron overload lead to insulin resistance. Plasma insulin levels rose excessively after oral glucose administration in the pubertal subjects with thalassemia compared to controls indicating increased insulin secretion developing in older children with thalassemia treated with long-term hypertransfusion therapy which later on results in insulin resistance [20,21]. A multicenter cross-sectional study conducted in Thailand in patients with thalassemia aged ≥18 years old concluded that iron overload is a statistically significant risk factor for DM in patients with TDT (AOR = 6.2, 95%CI (1.2-30.8), p-value = 0.02) [22]. Storage of whole blood under conventional blood bank conditions leads to significantly increased nonenzymatic glycosylation of Hb, plasma, and erythrocytic proteins [23]. 

There has been substantial research on the effect of hyperglycemia on the lifespan and metabolic properties of RBC but very little research on the effect of high sugar content of RBC storage media in blood banks. We therefore may describe RBC stored for long duration as “quasi-diabetic” [24]. We need to have a focused research on the relation between high sugar concentration in storage medium and changes in RBC functional properties during storage. Marked discrepancy between HbA1c and plasma FBS levels in this study has prompted consideration that the HbA1C assay used may not be reliable in TDT participants.

A significant difference was found in the BMI, RBC count, Hb, PCV, MCV, and MCHC values between controls and cases. Since thalassemia leads to the destruction of RBC, RBC count, Hb, PCV, MCV, and MCHC values are less in TDT patients in relation to age- and sex-matched controls. Low MCV acts as the first indication of this problem. Though the Mentzer index is not less than 13, this may be because the TDT participants are on continuous routine blood transfusion therapy. Moreover, because of repeated blood transfusions, associated complications, and slow growth rates, these patients fail to thrive to their normal potential, and hence, their BMI is also less than normal. The diagnosis and other detailed information on thalassemia have been given at the National Library of Medicine, and our study shows similar results [25].

A negative correlation was seen within the TDT group between HbA1c and Hb concentration in our study. A weak correlation was seen between FBS and HbA1c levels in TDT patients and between age and HbA1c levels. Advanced age statistically showed a weak correlation with higher HbA1c levels than those with normal glucose tolerance. A study in Korea found that lower Hb levels were associated with higher HbA1c levels at given fasting glucose levels. This result was consistent across fasting glucose quintiles, even in the normal population [26]. This study also demonstrated a positive association between HbA1c and fasting plasma glucose. Our study revealed a weak to moderate correlation between HbA1c and FBS.

Limitations

In TDT, frequent blood transfusions can lead to the presence of multiple types of Hb, impacting the reliability of HbA1c measurements for assessing glycemic control. Regular transfusions of RBCs can affect RBC turnover and lead to changes in HbA1c values, requiring further research. Iron overload can impact glucose metabolism and complicate the interpretation of HbA1c results. The small sample size may limit the generalizability of findings.

## Conclusions

Our study revealed a high prevalence of impaired glucose metabolism in TDT patients based on HbA1C levels. Early and accurate diabetes diagnosis is crucial, as these patients may have high blood sugar without obvious symptoms, increasing their risk of long-term complications and mortality. The elevated HbA1c in many TDT patients is concerning, especially since it often doesn't align with FBS levels. While HbA1c is the gold standard for diagnosing diabetes, caution is needed in interpreting these levels for TDT patients. In cases where HbA1c and blood sugar levels conflict, plasma glucose criteria should guide diabetes diagnosis. Additionally, alternative indicators like fructosamine, glycated albumin, and continuous glucose monitoring should be considered.
